# Risk of stroke-specific mortality after radiotherapy in patients with primary brain tumours

**DOI:** 10.1016/j.ctro.2023.100658

**Published:** 2023-07-06

**Authors:** Omar Kouli, Philip McLoone, David Morrison, Nicholas G. Zaorsky, Anthony J. Chalmers

**Affiliations:** a NHS Greater Glasgow and Clyde, Glasgow, UK; b School of Health and Wellbeing, University of Glasgow, UK; c Department of Radiation Oncology, University Hospitals Seidman Cancer Center, USA; d Case Western Reserve School of Medicine, Cleveland, OH, USA; e School of Cancer Sciences, University of Glasgow, UK

**Keywords:** Glioma, Radiotherapy, Stroke-specific mortality, Stroke, Cerebrovascular disease

## Abstract

**Introduction:**

Stroke is an established complication in cancer patients, amongst whom brain tumour patients have the highest risk of fatal stroke. Radiotherapy is an important treatment for brain tumours and is associated with increased risk of cerebrovascular disease. However, the impact of brain irradiation on stroke-related deaths in brain tumour patients is unknown, and the timing of any effect uncertain. This study investigates the relationship between radiotherapy and stroke-specific mortality (SSM) in patients with primary brain tumours.

**Methods:**

Patients of any age diagnosed with histologically confirmed primary brain tumours between 1992 and 2015 were abstracted from the Surveillance, Epidemiology, and End Results (SEER) database. Primary outcome was impact of radiotherapy on 5-year SSM. Cumulative SSM rates under competing risk assumptions were estimated and stratified by intervention type. Time-dependent hazard ratios were estimated to identify when the radiotherapy impact was greatest.

**Results:**

85,284 patients with primary brain tumour diagnoses were analysed. Overall, the 5-year cumulative SSM rate was low (0.6%) with the highest rate (0.76%) in patients receiving no treatment, in whom it mainly occurred < 1 month after diagnosis. SSM rates were lower in patients treated with radiotherapy alone (0.27%) or radiotherapy plus surgery (0.24%); stroke-related deaths also occurred later in these groups. While these patterns were observed in both glioblastoma and non-glioblastoma patients, stroke deaths tended to occur later in non-glioblastoma patients receiving radiotherapy. Relative to the ‘no treatment’ group, the highest risk of stroke mortality in radiotherapy treated patients occurred 3.5–4 years after diagnosis.

**Conclusion:**

The risk of SSM is low in patients with primary brain tumours and is not increased by radiotherapy. Two different patterns were observed: acute stroke mortality in patients receiving no treatment, and delayed stroke mortality in patients receiving radiotherapy (+/- surgery) with the latter peaking 3.5–4 years after diagnosis.

## Highlights

• The risk of stroke-specific mortality in patients with gliomas is low (approximately 0.6%).
• This risk is not increased in patients treated with radiotherapy.
• Acute stroke-specific mortality is more commonly observed in patients who do not receive radiotherapy.
• Patients treated with radiotherapy show a gradual increase in risk of death from stroke which peaks 3.5–4 years after diagnosis.

## Introduction

Primary brain tumours are a heterogenous group of neoplasms arising from cells within the central nervous system (CNS), amongst which gliomas have the highest incidence [1]. According to the Central Brain Tumour Registry of the United States [2], the average annual age-adjusted incidence rate for all CNS tumours is 23.4 per 100,000 population (7.1 for malignant vs 16.3 for non-malignant). Glioblastoma (GBM) is the most common malignant CNS tumour, accounting for 14.6% of all tumours and 48.3% of malignant tumours. Five year relative survival rates are much lower for malignant tumours than their non-malignant counterparts (35% vs 91%) with GBM having the worst outcomes (median survival 8 months and 5 year survival rate around 5%) despite the use of multimodal therapy comprising surgery, radiotherapy, and chemotherapy [2], [3].

Stroke, caused by cerebrovascular disease, is the fifth leading cause of overall mortality in the United States [4] and contributes to mortality in cancer patients. A recent study of 7.5 million cancer patients reported stroke-specific mortality (SSM) to be highest in patients with brain tumours [5]. Stroke in cancer patients can be caused by mechanical compression of blood vessels by tumours, via cancer-associated hypercoagulopathies or as a consequence of treatment [6]. As a complication of surgery, stroke generally occurs acutely whereas radiation effects on the vasculature are observed months or even years after treatment [6]. Radiotherapy damages blood vessels of all sizes, with arteries and capillaries being particularly sensitive [7]. Radiation induced endothelial cell death may be observed within hours or days and is often followed by thrombus formation and haemorrhage. In the brain, disruption of the blood–brain barrier exacerbates these effects by causing vasogenic oedema and localised hypoxia [7]. Over subsequent months and years this damage leads to endothelial proliferation, fibrosis and vascular dilatation, all of which are likely to increase the risk of stroke.

Previous studies have shown that stroke rates are increased in cancer patients receiving radiotherapy, with elevated risk particularly associated with radiation therapy to the head and neck area, including the brain [8]. To our knowledge, however, the impact of brain irradiation on risk and timing of stroke in patients with primary brain tumours has not been documented. In this study, we aimed to estimate the risk of death from stroke amongst patients with primary brain tumours, and to evaluate whether this risk was associated with radiotherapy treatment.

## Methods

### Study design and data sources

A retrospective, observational study was conducted according to the Strengthening the Reporting of Observational Studies in Epidemiology (STROBE) guideline [9]. Data collected from the population-based, prospectively maintained Surveillance, Epidemiology, and End Results (SEER) database was utilized in this study. The SEER program aims to deliver information on cancer statistics by providing population-based incident cancer registries, covering 35% of the US population, including incidence, survival, and intervention data [10].

### Study population

Patients of any age diagnosed with histologically confirmed primary brain tumours between 1992 and 2015 were retrospectively abstracted (diagnostic confirmation = “Positive histology” or “Pos hist AND immunophenotyping AND/OR pos genetic studies”). Patients without histological confirmation were excluded. The International Classification of Diseases for Oncology third edition (ICD-O-3) [11] coding system was utilised to group patients into two histological subgroups (non-GBM vs GBM (ICD-O-3 = “9440/3”)).

### Outcomes

Cause of death was categorized by the International Classification of Diseases (ICD)-10 code. The primary outcome measure was to evaluate the impact of radiotherapy on 5-year SSM rates defined as death due to stroke (specific cause of death = “Cerebrovascular Diseases”, ICD-10 = “I60-I69”).

### Statistical analysis

Continuous variables were summarised by mean (standard deviation), with appropriate parametric tests performed. Categorical data were expressed as counts and percentages, with differences tested using chi-square test. Survival durations were converted to number of years by months/12. Cumulative curves of SSM rates under competing risk assumptions were estimated for cumulative probability of mortality by intervention type (No treatment vs Radiation only vs Surgery only vs Surgery + Radiation). Subgroup analysis was undertaken to investigate cumulative SSM rates in GBM vs non-GBM tumours. Chemotherapy was not included in the ‘intervention type’ variable as the study’s aim was not to compare different intervention types but to evaluate the effect of radiotherapy on SSM in terms of magnitude and timing. However, to account for possible effects of chemotherapy, it was included as a covariate in the models built. Multivariable Cox proportional hazard analysis for SSM included variables such as age, gender (Male vs Female), race (American Indian/Alaska Native vs Asian or Pacific Islander vs Black vs White), chemotherapy (Yes vs No), GBM (Yes vs No) and intervention type. To account for the possibility that the outcome of interest (death due to stroke) may not have occurred because mortality from other causes happened first, a competing regression analysis was performed [12]. Because we hypothesised that the effect of radiotherapy on SSM might change with time, hazard ratios were split into time periods and presented as a time-dependent hazard ratio plot. All tests were two-sided with a significance level considered as p < 0.05. Effect estimates were summarized as odds ratios (OR) or hazards ratios (HR) with 95% confidence intervals (95% CI). All statistical analyses were performed using the RStudio graphical interface v.1.4 for R software environment v.4.0 [13].

## Results

**Fig. 1:**
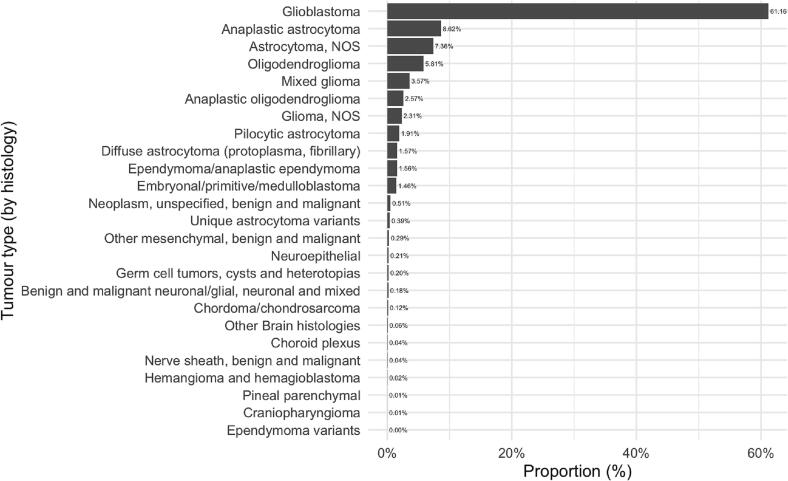
Breakdown of tumour types (by histology) included in analyses.

Overall, 99,349 cases of primary brain tumours were abstracted from the SEER database. 85,284 cases had positive histology and were included in the final analyses. Patients were aged between 0 and 103 years. 46,609 (54.6%) cases were diagnosed with GBM; other histological subtypes are shown in Fig. 1. Most patients were treated with a combination of surgery and radiotherapy (51.9%, n = 44324). One quarter of patients received surgical intervention only (25.8%, n = 22024), 13.5% (n = 11492) received radiotherapy only and 8.7% (n = 7444) had no treatment. Across all tumour types, the 5-year mortality rate was 71.4% (n = 60909). The most common cause of death (83.4%) was ‘Malignant Neoplasm of Brain and Other Parts of CNS’ (ICD-10= “C70, C71, C72”). In contrast, death was attributed to stroke in only 0.6% of cases (n = 359). Table 1 presents an overview of this cohort stratified by mortality cause.

**Table 1:** Overview of 85,284 patients diagnosed with Brain Tumours (Surveillance, Epidemiology, and End Results, 1992–2015).

		**Mortality**			
		**Alive**	**Died of Stroke**	**Died of Other causes**	**p-value**
Age (years)	Mean (SD)	33.6 (21.4)	59.6 (18.2)	58.1 (18.4)	<0.001
Race	American Indian/Alaska Native	166 (0.7)	2 (0.6)	267 (0.4)	<0.001
	Asian or Pacific Islander	1633 (6.7)	14 (3.9)	2693 (4.4)	
	Black	1787 (7.3)	39 (10.9)	3618 (6.0)	
	White	20,789 (85.3)	304 (84.7)	53,972 (89.1)	
Sex	Female	11,119 (45.6)	155 (43.2)	25,366 (41.9)	<0.001
	Male	13,256 (54.4)	204 (56.8)	35,184 (58.1)	
GBM	No	19,654 (80.6)	198 (55.2)	18,823 (31.1)	<0.001
	Yes	4721 (19.4)	161 (44.8)	41,727 (68.9)	
Chemotherapy	No	13,580 (55.7)	274 (76.3)	31,149 (51.4)	<0.001
	Yes	10,795 (44.3)	85 (23.7)	29,401 (48.6)	
Intervention	None	1469 (6.0)	69 (19.2)	5906 (9.8)	<0.001
	Radiation only	1482 (6.1)	36 (10.0)	9974 (16.5)	
	Surgery only	10,190 (41.8)	121 (33.7)	11,713 (19.3)	
	Surgery + Radiation	11,234 (46.1)	133 (37.0)	32,957 (54.4)	

SD, Standard Deviation; GBM, Glioblastoma.

**Fig. 2:**
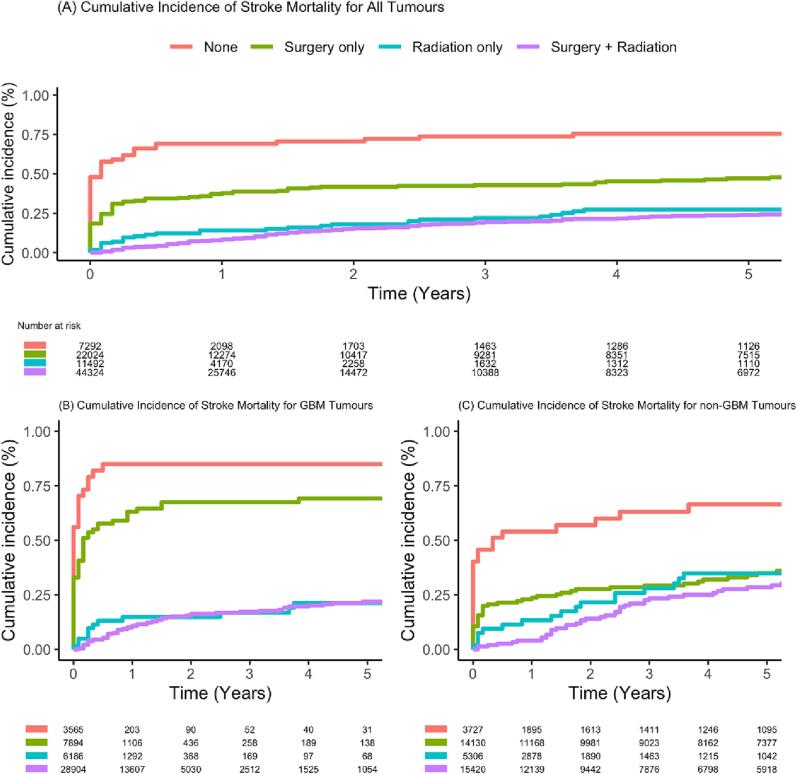
Cumulative incidence plots depicting 5-year stroke-specific mortality (from time of diagnosis) in all tumours (A), GBM (B), and non-GBM tumours (C).

Five-year cumulative SSM rates were lower in patients who had received radiotherapy (Fig. 2). Considering all tumours together, the highest 5-year SSM rate was observed in the ‘no treatment’ group (0.76%) compared with 0.47% for patients treated with surgery only, 0.27% for radiotherapy alone and 0.24% for radiotherapy plus surgery (Fig. 2A). Stroke deaths also occurred much earlier in the ‘no treatment’ group with a median of < 1 month from the time of diagnosis, compared with 1.5 years (IQR 0.25 to 35) and 2 years (IQR 0.88 to 6.0) in patients receiving radiotherapy alone or radiotherapy plus surgery, respectively.

In the subgroup analysis, SSM cumulative incidence rates for GBM and non-GBM tumours (Fig. 2B and 2C, respectively) exhibited similar trends, although stroke mortality occurred earlier in GBM patients. Within the GBM group, patients receiving no treatment had a 5-year incidence rate of 0.85%, while those treated with surgery only, radiotherapy alone, and radiotherapy plus surgery had lower incidence rates of 0.69%, 0.21%, and 0.31%, respectively. In the non-GBM group, patients receiving no treatment had a 5-year SSM rate of 0.66%, while those treated with surgery only, radiotherapy alone, and radiotherapy plus surgery had lower incidence rates of 0.35%, 0.35%, and 0.28%, respectively. Stroke deaths in both ‘untreated’ subgroups occurred very early after diagnosis (median < 1 month). Considering only patients receiving radiotherapy, stroke deaths occurred later in non-GBM than GBM patients, with median times of 2.4 vs 0.29 years (radiotherapy alone) and 4.7 vs 1.0 years (radiotherapy plus surgery) from time of diagnosis.

In a competing risk regression analysis predicting SSM (Table 2), increasing age was associated with increased risk of stroke mortality (OR: 1.03 per year, 95% CI: 1.03–1.04, p < 0.001) whereas a diagnosis of GBM (OR: 0.43, 95% CI: 0.33–0.55, p < 0.001) and radiotherapy only treatment (OR: 0.51, 95% CI: 0.34–0.77, p = 0.001) were associated with lower risk of stroke mortality.

**Table 2:** Competing risk regression predicting stroke-specific mortality (SSM).

		**All**	**HR (SSM CPH univariable)**	**HR (SSM CPH multivariable)**	**HR (competing risks multivariable)**
Age (years)	Mean (SD)	51.1 (22.3)	1.06 (1.05–1.07, p < 0.001)	1.06 (1.05–1.07, p < 0.001)	1.03 (1.03–1.04, p < 0.001)
Race	American Indian/Alaska Native	435 (0.5)			
	Asian or Pacific Islander	4340 (5.1)	0.79 (0.18–3.50, p = 0.761)	0.65 (0.15–2.87, p = 0.572)	0.67 (0.15–2.94, p = 0.600)
	Black	5444 (6.4)	1.79 (0.43–7.44, p = 0.420)	1.60 (0.39–6.65, p = 0.516)	1.48 (0.36–6.11, p = 0.590)
	White	75,065 (88.0)	1.09 (0.27–4.38, p = 0.903)	0.72 (0.18–2.91, p = 0.650)	0.73 (0.18–2.91, p = 0.660)
Sex	Female	36,640 (43.0)			
	Male	48,644 (57.0)	1.04 (0.84–1.29, p = 0.702)	1.19 (0.96–1.47, p = 0.112)	1.09 (0.88–1.35, p = 0.420)
Glioblastoma	No	38,675 (45.3)			
	Yes	46,609 (54.7)	1.86 (1.45–2.37, p < 0.001)	1.09 (0.83–1.43, p = 0.556)	0.43 (0.33–0.55, p < 0.001)
Chemotherapy	No/Unknown	45,003 (52.8)			
	Yes	40,281 (47.2)	0.40 (0.31–0.51, p < 0.001)	0.48 (0.36–0.63, p < 0.001)	0.53 (0.41–0.70, p < 0.001)
Intervention	None	7444 (8.7)			
	Radiation only	11,492 (13.5)	0.38 (0.25–0.57, p < 0.001)	0.48 (0.32–0.73, p = 0.001)	0.51 (0.34–0.77, p = 0.001)
	Surgery only	22,024 (25.8)	0.39 (0.29–0.53, p < 0.001)	0.67 (0.49–0.92, p = 0.013)	0.97 (0.70–1.34, p = 0.830)
	Surgery + Radiation	44,324 (52.0)	0.28 (0.21–0.38, p < 0.001)	0.46 (0.33–0.64, p < 0.001)	0.71 (0.51–1.00, p = 0.052)

HR, Hazard Ratio; CPH, Cox-Proportional Hazard; SSM, Stroke-Specific Mortality.

The time-dependent hazard plot (Fig. 2), in which hazard ratios were split into six-month periods, reveals that, relative to the no treatment group, the risk of stroke mortality peaked at different times in the different intervention groups. In the radiation only group, the peak hazard occurred 3.5 years after diagnosis while for the surgery + radiation group it occurred slightly later at 4 years.

## Discussion

In a recent population-based study using nationally representative data from SEER, Zaorsky et al. [5] found that the risk of stroke in cancer patients was twice as high as that of the general population. Specifically, the risk of fatal stroke was highest among patients with brain tumours, with a stroke-specific standardized mortality ratio of 7.63 (95% CI 5.66, 10.5, relative risk p < 0.0001) in the first 5 years following diagnosis. Investigating this observation in greater depth, however, we found that the overall risk of death from stroke in patients with primary brain tumours was low and constituted only 0.6% of all causes of death. In an adjusted competing regression analysis, increasing age was found to contribute to higher odds of SSM. On the other hand, patients with GBM had lower odds of SSM, probably because patients with this aggressive tumour experience short survival times [2]. We also made the important observation that patients treated with radiotherapy (alone or after surgery) had lower odds of SSM than those treated with surgery alone or receiving no treatment. This indicates that the known effects of radiotherapy on brain tissues including the vasculature very rarely manifest as fatal stroke.

Early deaths from stroke (median < 1 month) were observed more frequently in the group of patients who did not go on to receive further treatment (surgical resection or radiotherapy). We speculate that these early deaths were likely to have occurred in patients whose tumours became apparent because of a severe stroke, or who suffered a severe stroke in the immediate post-operative period. In either case, the stroke is likely to have contributed to the ‘no treatment’ decision. This pattern was observed in both GBM and non-GBM subgroups (Fig. 2B and 2C, respectively). Stroke deaths in patients receiving radiotherapy (+/- surgery) tended to occur much later than in patients who did not receive radiotherapy, especially in the non-GBM group. The most likely explanation is that patients with non-GBM glioma tend to survive longer than their GBM counterparts, giving more time for the adverse effects of radiotherapy on the brain to emerge. The 5-year cumulative SSM incidence in the radiotherapy alone group was higher in non-GBM patients than in the GBM group (0.35% vs 0.21%).

To our knowledge these are the first published data describing the impact and timing of radiotherapy on risk of stroke in patients with primary brain tumours of all grades. In 2015, Aizer and colleagues used the SEER database to study the risk of fatal stroke in patients with primary brain tumours excluding glioblastoma [14]. This study analysed data from 19,565 patients diagnosed between 1983 and 2002 and divided them into two groups: those with supratentorial tumours (12257 cases) and those with tumours involving the brainstem or infratentorial fossa (7308 cases). Consistent with our findings, they observed radiotherapy to be associated with reduced risk of fatal stroke in patients with supratentorial tumours. In the other group, however, radiotherapy was associated with an increased risk, which the authors attributed to radiation effects on the central vasculature of the brain [14]. The group with infratentorial or brain stem tumours is likely to have included a higher proportion of children (data not available in the paper), and there is clear evidence that brain irradiation as part of cancer treatment for children significantly increases their risk of subsequent stroke [15], [16], [17]. In the paediatric population, higher total radiation dose [16] and irradiation of major cerebral arteries have been reported to associate with higher risk [15], [17], [18]. Median times from diagnosis to stroke were in the region of 7–9 years in the paediatric studies, most of which had access to long term follow up data (up to 25 years in some cases). Treatment related effects are likely to be easier to detect in children, who would otherwise be at extremely low risk of stroke. Previous studies of patients with pituitary adenoma have reported increased incidence of stroke [19], [20] and cerebrovascular mortality [21], [22] in these patients compared with the general population, but provide no evidence that radiotherapy exacerbates the risk of stroke or SSM.

**Fig. 3:**
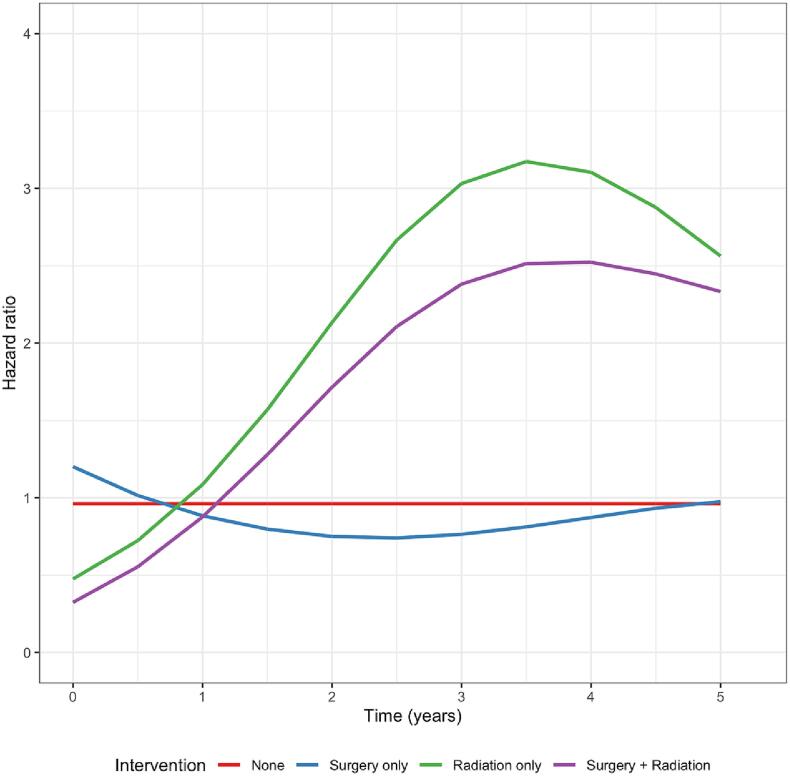
Time-dependent hazard ratio plot for intervention types (No treatment “None” was used as reference group).

A recent *meta*-analysis of nearly 58,000 cancer patients reported a 2.09-fold higher risk of stroke in those receiving radiotherapy, with a marginally higher relative risk of 2.16 in patients receiving radiotherapy to the brain or head and neck regions [8]. Radiation-induced stroke is a complex pathophysiological process which evolves over time, as outlined in the introduction. Time of onset is thought to be dependent on radiation dose: after low doses, vascular injury is not immediately evident and infarcts may develop many years after exposure [7]. In our study, we found that patients treated with radiotherapy (+/- surgery) exhibited a gradual increase in risk of death from stroke (Fig. 1) with the highest relative hazard risk at around 3.5–4 years (Fig. 3). This finding supports the hypothesis that radiotherapy exposure to the brain is associated with a delayed risk of stroke and associated mortality, but indicates that the absolute risk is low. It is possible, however, that the risk will continue to rise in patients surviving beyond five-year follow-up period studied here.

This study has many limitations. The SEER database lacks detail regarding stroke subtypes (haemorrhagic vs ischaemic) and other risk factors that increase risk of stroke including co-morbidities and laboratory biomarkers. It is also highly likely that the stroke is under-reported as the cause of death in this population of patients who are known to have primary brain tumours. And while few of the patients included in this study are likely to have received bevacizumab as part of the oncological treatment, this anti-angiogenic agent that is known to be associated with increased risk of ischaemic stroke [23] is now more widely used and may impact on radiotherapy effects in the future. Coupled with the very low SSM rates observed (0.6% of causes of death in brain tumours), these limitations render extended analyses to identify risk factors leading to increased SSM unfeasible. The results of our analysis are not intended to quantify and compare different effects of interventions and risk factors on SSM but to provide a general overview and pose questions for future investigation.

## Conclusion

The risk of SSM in patients with brain tumours is low (approximately 0.6%) and is not increased in patients treated with radiotherapy. Two different patterns are observed: acute mortality, which is likely to be associated with stroke as a presenting symptom or a complication of surgery; and delayed mortality, which may be associated with radiotherapy. In support of the latter statement, patients treated with radiotherapy (+/- surgery) show a gradual increase in risk of death from stroke which peaks 3.5–4 years after diagnosis.

## Patient consent statement

The anonymous clinical data analysed in this manuscript was obtained from the SEER database and patient consent was not required for its use.

## Source of funding

Cancer Research UK: Radiation Research Centre of Excellence at the University of Glasgow (C16583/A28803).

## CRediT authorship contribution statement

**Omar Kouli:** Conceptualization, Methodology, Formal analysis, Investigation, Visualization. **Philip McLoone:** Methodology, Formal analysis, Visualization. **David Morrison:** Conceptualization, Methodology, Supervision. **Nicholas G. Zaorsky:** Conceptualization, Methodology, Formal analysis, Data curation. **Anthony J. Chalmers:** Conceptualization, Supervision, Project administration, Funding acquisition.

## Declaration of Competing Interest

The authors declare that they have no known competing financial interests or personal relationships that could have appeared to influence the work reported in this paper.
